# Virtual Interactive 3D Modeling to Improve Outcomes in Robot-Assisted Partial Nephrectomy: Clinical Trial Protocol for the Multicenter, Randomized 3DPN Study

**DOI:** 10.1016/j.euros.2025.04.004

**Published:** 2025-05-19

**Authors:** Sigrun Holze, Clara Steiner, Anja Dietel, Toni Franz, Phuc Ho Thi, Nicole Köppe-Bauernfeind, Miroslav Bačák, Meinhard Mende, Jens-Uwe Stolzenburg

**Affiliations:** a Department of Urology, University Hospital Leipzig, Leipzig, Germany; b Lank Center for Genitourinary Oncology, Dana-Farber Cancer Institute, Boston, MA, USA; c Clinical Trial Center Leipzig, University of Leipzig, Leipzig, Germany

**Keywords:** Robot-assisted partial nephrectomy, Kidney mass, Minimally invasive surgery, 3D modeling, Nephron-sparing surgery, Renal cell carcinoma

## Abstract

3DPN is a prospective, multicenter, randomized, controlled, two-armed open trial with planned recruitment of 370 patients in ten centers over a period of 24 mo. The trial is evaluating the value of virtual interactive 3D modeling for robot-assisted partial nephrectomy (RAPN) by assessing clinical outcomes, patient understanding, and surgeon confidence. Patients aged ≥18 yr with kidney tumors ≤7 cm who have contrast-enhanced computed tomography (CT) scans available and for whom RAPN with a da Vinci Surgical System is planned are eligible for the study. Using 1:1 randomization, patients are assigned to either the 3D model–based RAPN arm or the 2D CT scan–based RAPN arm.

This trial is registered on ClinicalTrials.gov as NCT06056505.

## Take Home Message

The multicenter prospective randomized controlled 3DPN trial is investigating if virtual interactive 3D modeling improves outcomes of robot-assisted partial nephrectomy in comparison to standard-of-care computed tomography imaging. The study will assess clinical and oncological parameters, patient understanding, and surgeon confidence levels.

## 1 Introduction and hypotheses

Renal cell carcinoma (RCC) ranks among the most common urological cancers along with prostate and bladder cancer, with more than 14 000 new cases annually in Germany in the past few years. Approximately 60% of RCCs are diagnosed at an early stage suitable for curative surgical treatment [1]. Recent developments in robot-assisted surgery have changed the distribution of surgical approaches between open, classic laparoscopic, and robot-assisted procedures in favor of robotic surgery. For instance, results from the British Association of Urological Surgeons demonstrated an increase in robot-assisted partial nephrectomy (RAPN) from 0% in 2008 to ∼62% in 2017, when RAPN exceeded the number of both open and laparoscopic partial nephrectomy (PN) surgeries [2]. However, given the anatomic structure of the kidney, tumors of similar size may vary highly in the level of difficulty for surgical resection, depending on factors such as proximity to the hilum or the calyceal system and endophytic versus exophytic growth. Different scoring systems have been proposed to classify renal tumors and standardize risk assessment. Ficarra et al [3] introduced the Preoperative Aspects and Dimensions Used for an Anatomical (PADUA) classification of renal tumors, and their prospective validation showed significant correlation between overall complication rates and the anatomic characteristics included in the score. The RENAL nephrometry score published by Kutikov and Uzzo [4] uses a comparable approach for reproducible quantitation of the complexity of renal lesions in the preoperative planning phase. In both classification systems, higher scores indicate more complex tumors, which increase the degree of surgical difficulty and thereby potentially impact the total operating time.

Current guidelines underscore the high significance of organ preservation in RCC surgery, with a strong recommendation for PN for T1 tumors [5]. The trifecta concept summarizes desired outcomes of PN, including negative surgical margins, maximal preservation of renal function, and complication-free patient recovery [6]. Preoperative surgical planning for each individual case is essential to achieve these goals. In current clinical practice, 2D computed tomography (CT) or magnetic resonance imaging scans are used to visualize anatomic relationships, and implementation of innovative 3D techniques is lacking. One such technique uses image segmentation from medical scans for virtual interactive 3D modeling [7]. These 3D models provide a patient-specific visualization of all crucial anatomical structures, including vessels, the renal collecting system, and the tumor, as well as their relative location to each other. This improves perception of the spatial relationships, which would otherwise require cognitive reconstruction of the different CT imaging planes [8]. This concept is supported by work by Amparore and colleagues [9], who recently showed that assessment of the complexity of renal masses with the PADUA and RENAL nephrometry scores was more accurate using 3D models.

With a virtual interactive 3D model, the surgeon can plan the operation in detail, for example, by identifying the location of vessels infiltrating the tumor, setting a strategy for exploration of the renal pedicle, and evaluating the proximity of the tumor to the pelvicalyceal system. Throughout the operation, the 3D model facilitates spatial orientation within the surgical field and makes it easier to define resection margins by clearly outlining the depth of tumor growth in comparison to a conventional CT scan. These potential improvements in surgical planning and strategy lead to the hypothesis that the process is more streamlined and results in a shorter operative time at the robotic console, and may eventually improve RAPN outcomes.

Since virtual interactive 3D modeling is a fairly new approach and surgical clinical trials are challenging to conduct, the data available—especially from large, randomized, multicenter studies—are limited. However, there is some evidence that 3D modeling may be a valuable tool to improve crucial parts of the surgical process [8,10]. For example, Shirk et al [11] reported shorter operating time, lower blood loss, and shorter clamp time and length of hospital stay for cases in which 3D virtual reality models were used during operative planning for RAPN. Furthermore, a recent case-matched study using 3D virtual models for minimally invasive PN demonstrated better outcomes with a lower reduction in estimated glomerular filtration rate (eGFR) and lower postoperative complication rates than in the matched group [12].

The aim of 3DPN is to investigate the utility of virtual interactive 3D modeling in RAPN by comparing this approach to conventional CT scans in a multicenter randomized controlled trial (RCT).

## 2 Study design and protocol

3DPN is a prospective, two-armed, multicenter RCT. Ten centers are participating in the study: the lead center at the University of Leipzig; University Hospitals Dresden, Mainz, Magdeburg, Homburg, Mannheim, and Bonn; Sana Clinic Borna; Helios University Hospital Wuppertal; and St. Antonius Hospital Gronau.

**Fig. 1:**
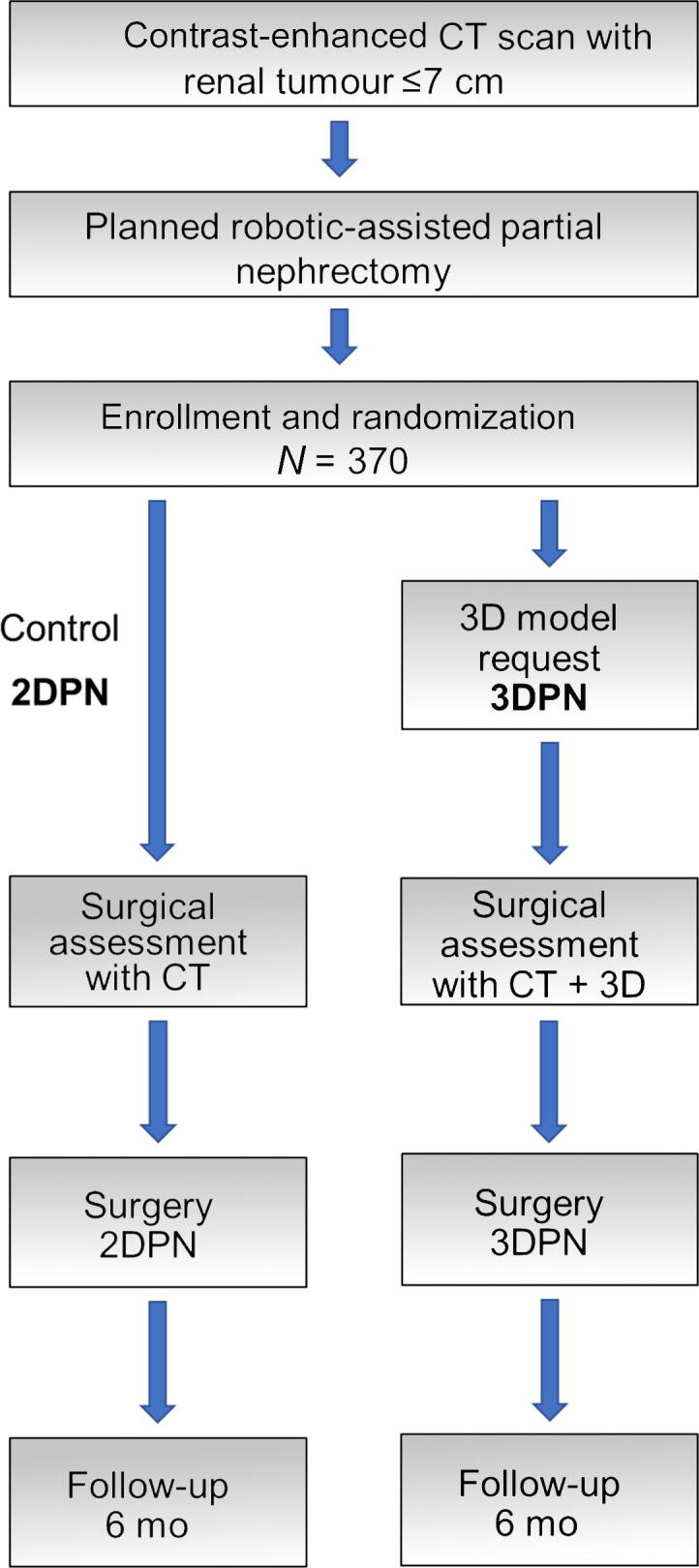
Study flow chart. CT = computed tomography; 2DPN = partial nephrectomy with 2D CT scans; 3DPN = partial nephrectomy with CT scans and 3D models.

**Fig. 2:**
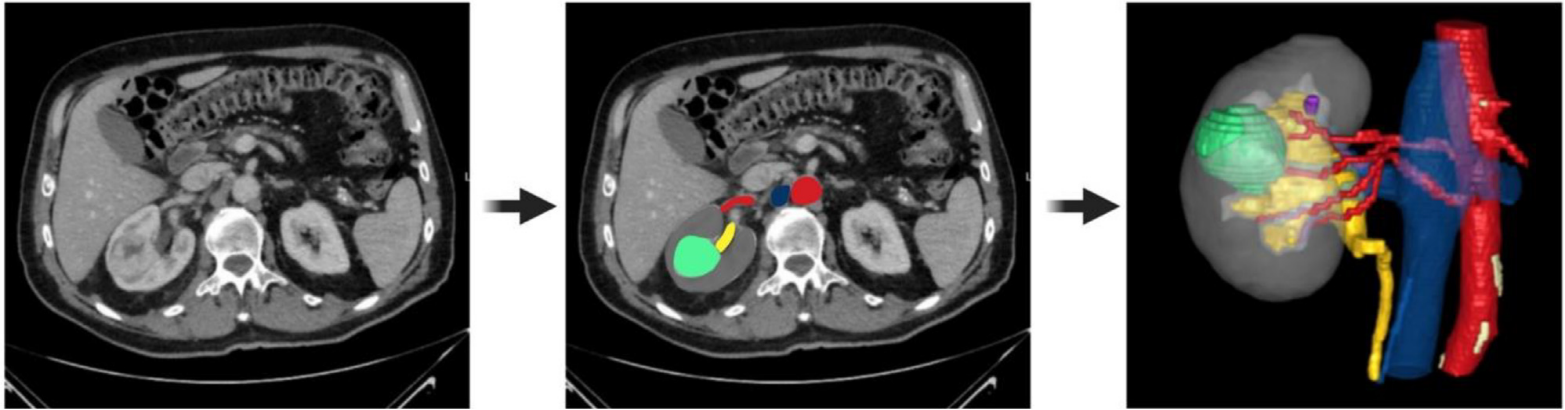
The 3D modeling process.

A total of 370 patients will be enrolled in the trial. Participants are randomized 1:1 to either the interventional arm, in which a 3D model is used in addition to conventional CT scans, or the control arm, in which only CT scans are used for preoperative and intraoperative planning and decision-making (Fig. 1). To balance the complexity of renal lesions and surgeries across both arms, patients are stratified according to the PADUA score for their renal mass (<8 vs ≥8) and body mass index (BMI; <35 vs ≥35 kg/m^2^) for randomization. The 3D models are available on a web-based application that allows interactive rendering on a tablet before and during the surgery. Innersight Labs (London, UK) generates the 3D models using DICOM data from CT scans for medical image segmentation (Fig. 2). Optimal 3D model quality is achieved with a slice thickness of ≤1.5 mm. One contrast-enhanced CT phase is needed for conceptualization of a 3D model. If multiple phases are available, the most suitable one is selected, typically with a focus on the arterial phase. However, the trial uses contrast-enhanced CT images from routine clinical care that are often part of the diagnosis for renal lesions and/or preparation for planned PN, and an arterial phase is not always available. Nevertheless, no additional in-house CT scan at the participating trial site is required. While the 3D model can also be created on the basis of an excretory phase in some cases if neither an arterial nor a venous phase is available, reconstruction of the renal pelvicalyceal system does not require an excretory phase.

Table 1 summarizes the data collection at four trial visits from baseline until follow-up 6 mo after surgery. Enrollment of the whole patient cohort is planned over a period of 24 mo. The trial opened in October 2023, with randomization of the first patient on October 24, 2023. After completion of follow-up, it is anticipated that results from the primary analysis of the trial will be available in the second quarter of 2026. The study is registered on ClinicalTrials.gov (NCT06056505).

**Table 1:** Schedule of assessments and procedures

Item		Visit 1 Baseline Enrollment and randomization	Visit 2 Surgery	Visit 3 Postoperative monitoring until discharge	Visit 4 Follow-up a 6 mo ± 8 wk after randomization
1	Inclusion and exclusion criteria	✓			
2	Patient informed consent	✓			
3	Randomization	✓			
4	Surgery consent b	✓			
5	Tumor characteristics	✓			
6	PADUA score	✓			
7	RENAL score	✓			
8	Oncological parameters c	✓		✓	
9	Comorbidities and medications (Charlson comorbidity index)	✓			
10	CT scan d	✓			
11	3D modeling e	✓			
12	Physical examination	✓			
13	Laboratory tests (blood count, creatinine, and eGFR)	✓		✓^f^	
14	Request for laboratory tests (creatinine and eGFR)				✓^g^
15	Urinalysis (albuminuria, hematuria)	✓			
15	Patient Reactions Assessment questionnaire h	✓			
16	Patient anxiety questionnaires (STOA, APAIS, NRS-A)	✓			
17	Patient satisfaction questionnaire	✓			
18	Surgeon confidence questionnaire		✓		
19	Adverse events		✓	✓	✓
20	Complications by Clavien Dindo grade		✓	✓	
21	Information on additional treatments				✓

APAIS = Amsterdam Preoperative Anxiety and Information Scale; CT = computed tomography; eGFR = estimated glomerular filtration rate; NRS-A = Numerical Rating Scale for Anxiety; STOA = State-Trait Operation Anxiety.

a Telephone interview with the patient by the study center in Leipzig (Department of Urology).

b With addition of a 3D model if the patient is randomized to the 3D model arm.

c May not be available until discharge.

d A contrast-enhanced CT scan of the patient’s kidney performed by an external physician is sufficient; the CT scan does not necessarily need to be performed at the study site.

e Only if the patient is randomized to the 3D model arm.

f According to local clinical routine, up to daily.

g Tests are requested via a fax form to the urologist in private practice, if available.

h All questionnaires are given to the patient after information on surgery is provided and consent is obtained, and before surgery is performed.

### 2.1 Eligibility criteria

Patients are only considered eligible for the 3DPN trial if all the following inclusion criteria are met:

• The patient has a kidney tumor ≤7 cm and RAPN with a da Vinci Surgical System is planned;
• A contrast-enhanced CT scan is available;
• The patient is aged ≥18 yr; and
• The patient has provided written informed consent.

The following exclusion criteria apply at the time of eligibility evaluation:

• History of surgery on the affected kidney (eg, PN, pyeloplasty, kidney cyst deroofing, percutaneous nephrolitholapaxy, radiofrequency ablation);
• Clinical suspicion of metastases (stage cN1/cM1);
• Horseshoe kidney;
• Multifocal kidney tumor;
• Von Hippel-Lindau syndrome and other hereditary kidney cancer syndromes;
• Previous malignancy with ongoing or planned nephrotoxic chemotherapy;
• Current immunosuppression (eg, organ transplantation, leukemia);
• Tumor thrombus in the renal vein or inferior vena cava;
• Severe cognitive impairment;
• Pregnancy, lactation, or a woman who wishes to have children;
• Patients under legal supervision or guardianship;
• Inability to give informed consent or suspected lack of compliance; and
• Patients who decline data collection and storage for the main study.

### 2.2 Primary objective

The primary objective is to determine whether 3D modeling reduces the total console operation time for RAPN as a surrogate endpoint for PN outcomes such as perioperative complications and morbidity.

### 2.3 Secondary objectives

Secondary endpoints include assessment of the patient benefit and aspects of treatment success and safety. These are categorized into three groups.

#### 2.3.1 Preoperative

• Questionnaires are used to evaluate patient anxiety (State-Trait Operation Anxiety, Amsterdam Preoperative Anxiety and Information Scale, Numerical Rating Scale for Anxiety), patient comprehension of the surgical condition and the procedure planned (Patient Reaction Assessment), and patient satisfaction.

#### 2.3.2 Intraoperative

• Hilar clamping technique (global ischemia, selective ischemia, clampless);
• Warm ischemia time (WIT) in minutes;
• Estimated blood loss;
• Number of transfusions;
• Total operative time measured from incision to suture in minutes;
• Number of conversions to open surgery (reported intraoperatively);
• Number of conversions to radical nephrectomy; and
• Surgeon confidence level evaluated via a questionnaire survey (Supplementary material; study-specific questions on the impact of imaging on the following topics: spatial orientation, surgeon confidence during the procedure, surgical strategy, visualization of anatomical structures, satisfaction with the imaging available).

#### 2.3.3 Postoperative

• Positive surgical margin rate;
• Creatinine and eGFR according to the Chronic Kidney Disease (CKD)-Epidemiology Collaboration equation during the hospital stay and at follow-up at 6 mo ± 8 wk after randomization; and
• Patient length of stay (recorded at discharge).

All secondary endpoints are exploratory, and no adjustment for multiplicity is foreseen. If the primary endpoint is not met, these objectives do not suffice to demonstrate the effectiveness of the tool, but may provide evidence suggesting its utility.

### 2.4 Complications and adverse events

The trial team assesses intraoperative complications and postoperative adverse events up to 6 mo after surgery and reports them as safety endpoints. The Clavien-Dindo classification scheme is used to grade postoperative complications.

### 2.5 Statistical analysis

Existing data are limited as virtual interactive 3D modeling is a recent innovation. However, taking into account current results in the literature, we aim to show a reduction in mean operation time by 15 min, assuming this decrease is clinically (and economically) relevant. Furthermore, we propose a standard deviation for the operation time of 50 min. To detect the difference in operation time using a two-tailed Mann-Whitney *U* test, a sample size calculation via simulation using PASS 2008 software (NCSS LCC, Kaysville, UT, USA) resulted in 185 patients per group, assuming power of 80% and a significance level of 5%. No missing values are expected because the operation time can be assessed for every patient treated. We anticipate dropouts at later visits and will account for missing values for secondary endpoints using mixed models. To gain further insight into the data obtained, analyses will be conducted for three different study populations. (1) The full analysis set will include all randomized patients, with analysis according to their allocated trial arm; those for whom 3D models are missing by the time of surgery will remain in the 3D group. This set corresponds to the intention-to-treat principle in the International Council for Harmonization Good Clinical Practice guidelines. (2) The per-protocol set will consist of all patients undergoing RAPN without major protocol violations that affect the primary endpoint. Major violations include failure to acquire 3D models in time for surgery planning (excluded in this population). (3) The safety analysis set will include all patients for whom surgery was planned using a 3D model. Further analyses will include (mixed) linear models, logistic regression, and descriptive statistics.

### 2.6 Data management and protection

Once patient eligibility is confirmed and informed consent to trial participation and data collection is obtained, the patient is assigned a unique patient identification code. Patient identification code lists are part of the investigator site file and remain at the recruiting site. These lists are the only documents that allow for reidentification of patients and can only be accessed by authorized members of the 3DPN study group. Clinical data are entered into electronic case report forms in a pseudoanonymized way using only the patient’s identification code. Data are collected at predefined time points, as summarized in Table 1.

The secuTrial database being used is protected against unauthorized access and validated according to standard operating procedures. The servers are physically located at Clinical Trial Center Leipzig (ZKS) in access-protected server rooms. All relevant trial documentation will be stored for 10 yrs after the end of the trial. After the archiving period, clinical data will be anonymized and stored for future use.

### 2.7 Ethics and administration

The ethics committee of the primary study center in Leipzig (Ethik-Kommission der Medizinischen Fakultät Leipzig) granted ethical approval for the study (reference 185/23-ek), followed by all participating study centers. The first amendment of the trial was approved in January 2025 authorizing the current state of the protocol as outlined here.

### 2.8 Trial supervision

ZKS is responsible for trial monitoring, including initiation, regular, and closeout visits at all trial sites. A risk-based monitoring strategy was implemented, as required by DIN ISO 14155 Chapter 6.7. During trial conduct, central and statistical monitoring procedures are combined with on-site monitoring visits to ensure high protocol compliance and data quality, and to guarantee patients’ rights and safety.

In addition, audits by an unbiased auditor are planned to guarantee trial compliance with DIN ISO 14155 and European and national laws.

## 3 Discussion

Owing to the complexity of the renal anatomy, visualization of anatomic relations for PN can be challenging in terms of vascular supply and tumor localization despite multiplanar and thin-slice CT scans. The advent of innovative tools for 3D imaging in preoperative planning and intraoperative navigation holds the potential to improve these crucial steps. Piramide et al [10] summarized the existing evidence for 3D modeling in minimally invasive PN in a 2022 systematic review and meta-analysis. Among the nine RAPN studies included in the review, only the trial by Shirk and colleagues [11] was designed as an RCT and enrolled 92 patients. To date, the 3DPN study is the largest RCT in the field and is assessing the effects of virtual interactive 3D models on total console operation time, perioperative and postoperative outcomes, psychological parameters at the patient level, and surgeon confidence.

The choice of the primary endpoint was based on the literature on RAPN. For example, Kim et al [13] showed a ∼20% reduction in console operation time in comparison to the matched control group, while there was no significant difference in WIT. Furthermore, 3D modeling might particularly be helpful for renal hilum preparation and tumor localization, neither of which would be reflected by WIT. Achieving a shorter operation time would automatically lead to a shorter anesthesia time, which is associated with a lower risk of complications [14]. Previous studies revealed a higher complication rate for longer operation times, especially for thromboembolism [15] and infections [16,17] in both general and urological surgeries. In the PN setting, a recent matched case-control study showed that after adjustment for covariates, virtual 3D modeling reduced the estimated average operation time from 201 min to 143 min [18].

Another advantage of 3D reconstruction is the potential to avoid radical nephrectomy in some cases. In an exploratory study of PN cases in which 3D modeling was retrospectively added to conventional CT diagnostics, indications for PN increased by 27% after surgeons reviewed the 3D model in addition to the CT scan [19]. Higher rates of organ preservation reduce the number of patients with CKD or other chronic conditions, with a consequent reduction in the number of hospital visits. The 3-yr probability of freedom from new-onset CKD was 35% after radical nephrectomy versus 80% after PN [20]. In addition, patients undergoing PN had a lower incidence of cardiovascular events in comparison to RN [21]. These factors can lead to higher quality of life and longer life expectancy with PN while maintaining oncologic control.

At the patient level, 3D modeling could facilitate patient understanding and improve overall satisfaction [22]. The SPLICE prospective RCT investigated an informed consent process involving 3D virtual reality for patients undergoing intracranial tumor resection. The results revealed higher objective comprehension of their condition for patients in the 3D group, while subjective comprehension and anxiety levels did not differ significantly between the study groups [23]. The 3DPN trial aims to investigate corresponding effects in the specific setting of PN.

Recent literature also supports the benefits of 3D reconstruction for surgeons. Better visualization of anatomical structures can lead to more informed decision-making about the operative approach, and modification of the surgical strategy after reviewing the 3D model in some cases [24,25]. Exploratory analyses will focus on the impact of visualization and surgeon confidence during PN.

As the 3DPN study follows standard-of-care treatment for renal tumors up to 7 cm and adds 3D images as a supplementary tool to standard CT imaging, no added risk is anticipated in the interventional arm. Furthermore, scans that are already available are used to render the 3D model, and no further invasive tests or added exposure to radiation are necessary.

Despite its strengths, the study has a few limitations. For instance, the endpoints being investigated could be influenced by the level of surgeon experience. To limit this performance bias, participating surgeons need to demonstrate a minimum of 100 previous robotic kidney surgeries. Moreover, patient stratification is based on BMI and the PADUA classification of renal tumors as a surrogate for tumor complexity. Unfortunately, not all renal lesions fit the schema of the anatomical criteria included, and scores can underestimate or overestimate the risk.

In summary, our goal is to demonstrate the utility of virtual interactive 3D modeling in a multicenter randomized clinical trial in ten high-volume tertiary care hospitals in Germany that perform PN regularly. We want to generate strong clinical evidence that could guide current PN practice in Germany and worldwide.

  ***Conflicts of interest***: The authors have nothing to disclose.

  ***Funding support****:* This study is funded by Deutsche Krebshilfe (German Cancer Aid; grant number 70114653).
