# Supplementary material for: Virtual Interactive 3D Modeling to Improve Outcomes in Robot-Assisted Partial Nephrectomy: Clinical Trial Protocol for the Multicenter, Randomized 3DPN Study
Source: Eur Urol Open Sci. 2025 May 19;76:58–64. doi: 10.1016/j.euros.2025.04.004 (PMC12148422; doi:10.1016/j.euros.2025.04.004)
Supplement: Supplementary Data 1 [file mmc1.docx]

**Supplementary Material: Questionnaire survey assessing the impact of available imaging on the operating surgeon**
